# FraMaDySc: dysphagia screening for patients after surgery for head and neck cancer

**DOI:** 10.1007/s00405-023-07865-6

**Published:** 2023-02-14

**Authors:** Christiane Hey, Almut Goeze, Robert Sader, Eugen Zaretsky

**Affiliations:** 1 Department of Phoniatrics and Pediatric Audiology, Marburg University Hospital, University of Marburg, Marburg, Germany; 2 Oral, Cranio-, Maxillofacial and Facial Plastic Surgery, Frankfurt (Main) University Hospital, University of Frankfurt (Main), Frankfurt (Main), Germany

**Keywords:** Dysphagia, Swallowing disorder, Head and neck cancer, Postsurgical, Screening

## Abstract

**Purpose:**

Oropharyngeal dysphagia is one of the most common postoperative consequences in head and neck cancer patients. Above all, these patients often suffer from aspiration and limitations of oral intake. However, no reliable dysphagia screening is available for this target group. This study aimed to develop and validate a screening, *FraMaDySc*, based on a water swallow test (WST) for the identification of postsurgical patients with a risk of aspiration, limitations of oral intake, and, as their derivate, a relevant oropharyngeal dysphagia in general (OD) that constituted the main reference standard.

**Methods:**

A total of 184 postsurgical head and neck cancer patients were tested with a WST. The patients were, on average, 62 years old and predominantly male (71%). After WST, they underwent Fiberoptic Endoscopic Evaluation of Swallowing (FEES^®^). FEES^®^ results were dichotomized by Penetration aspiration scale and Functional oral intake scale. Patients with a “fail” result on one of these two scales were classified as having OD. Quality criteria of WST were quantified by means of cross-tabulation with FEES^®^ results.

**Results:**

OD was found in 65% of patients, aspiration in 44%, limitations of oral intake in 56%. WST delivered a “fail” result in 65% of patients. WST showed sensitivity 91% and specificity 88% for OD. Quality criteria for aspiration (sensitivity 64%, specificity 93%) and limitations of oral intake (sensitivity 80%, specificity 87%) were lower.

**Conclusion:**

*FraMaDySc* is a standardized, quick, and valid WST and therefore an excellent screening tool for the identification of OD in postoperative head and neck cancer patients.

**Supplementary Information:**

The online version contains supplementary material available at 10.1007/s00405-023-07865-6.

## Introduction

Despite advances in surgical treatment and reconstruction modalities, swallowing disorders are still one of the most critical postsurgical consequences in head and neck cancer patients [1, 2]. With up to 75% of this patient population, their prevalence is remarkably high [3] and varies depending on the tumor site and size as well as resection extent [4, 5]. Oropharyngeal dysphagia can cause substantial life-threatening complications such as aspiration pneumonia and malnutrition that are both associated with an increased morbidity [4, 6–8], length of hospital stay [9, 10], and tube dependency [11, 12]. Thus, early identification of dysphagia and its timely management are vital for the reduction of these critical postsurgical sequels both for patient’s health and healthcare system [13, 14].

However, instrumental diagnostics is time-consuming and costly, also in terms of personnel and other resources. Therefore, a valid and standardized screening tool is considered one of the most critical components in the clinical routines for detecting patients with a high risk of relevant oropharyngeal dysphagia. Still, there is a lack of such dysphagia screenings for head and neck cancer patients undergoing surgery.

In general, different screening approaches can be utilized for detecting patients at risk for swallowing impairments such as anamnestic data, clinical assessment, and water swallow tests (WST). Whereas anamnestic data and the examination of clinical findings like gag reflex, wet voice or volitional cough often failed in predicting dysphagia [15], some WST demonstrated excellent performance indices, at least in post-stroke patients [16–18].

Clinicians providing care to stroke patients focus on aspiration as the leading reference criterion in screening methods. However, swallowing disorders resulting from head and neck cancer differ considerably from other dysphagia etiologies. Due to vast oral transport impairments, a relevant oropharyngeal dysphagia can occur without any signs of aspiration but with limitations of oral intake, among other things [19]. Hence, a dysphagia screening for postsurgical head and neck cancer patients should be able to identify patients at risk of (a) aspiration, (b) limited oral intake and above all (c) a relevant oropharyngeal dysphagia (OD) in general that requires further management and care. Thus, this study aimed to develop and evaluate such a screening tool based on a WST for this particular patient population, with the relevant OD being the most important reference criterion because of its high importance in the everyday clinical practice.

## Patients and methods

This prospective study was performed between 2010 and 2022 in line with the principles of the Declaration of Helsinki. Approvals were granted by the Ethics Committees of Frankfurt/Main University Hospital (approval # 240/10) and Marburg University Hospital (approval # 63/15).

### Development of the water swallow test *FraMaDySc*

**Fig. 1:**
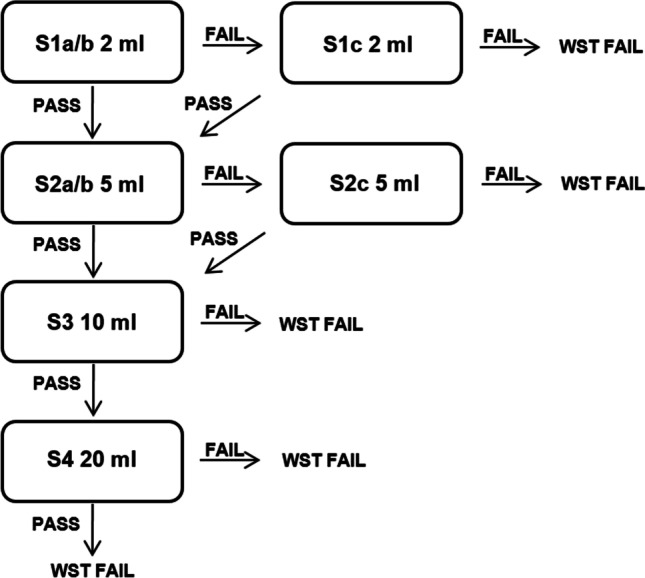
Flow chart of the water swallow test (WST)

Based on clinical experience and previous scientific research [20–22], a WST was constructed for the development of *FraMaDySc (Frankfurt/Marburg Dysphagia Screening)*. For safety reasons, this WST includes increasing calibrated volumes of water (2, 5, 10, and 20 ml) to minimize the risk of aspiration. The first volume (2 ml) is administered by a spoon, all other volumes by a plastic beaker. The two smaller volumes, 2 and 5 ml, are offered twice (a- and b-attempts), with an optional third attempt in case of failure (c-attempt). Two larger volumes, 10 and 20 ml, are given only once without any further attempts (see Fig. 1). The total amount of water is 44 ml if all swallow attempts are normal or 51 ml in case of the optional c-attempts.

Three WST failure criteria were defined: (a) wet voice before swallowing, (b) wet voice or voice change after swallowing, (c) cough or throat clearing after swallowing. If a patient demonstrates wet voice before the first swallow attempt (2 ml) without being able to clear, the WST is not performed and valued as a “fail”.

### Study sample

The WST was evaluated in head and neck cancer patients after tumor surgery including resection and, if necessary, plastic reconstruction. The inclusion criteria were (a) age 18 + years, (b) written informed consent, (c) Union for International Cancer Control (UICC) stages II–IV, (d) no pre-existing dysphagia, (e) no neurological diseases such as Parkinson’s disease, (f) no total laryngectomy. The sample size was calculated in the software BiAS 8 (Epsilon publishing house, Hochheim and Darmstadt, Germany) and amounted to *N* = 184 under condition of *α* = 0.05, power = 90%, and prevalence of swallowing disorders 75% in head and neck cancer patients after surgery (see Introduction).

**Fig. 2:**
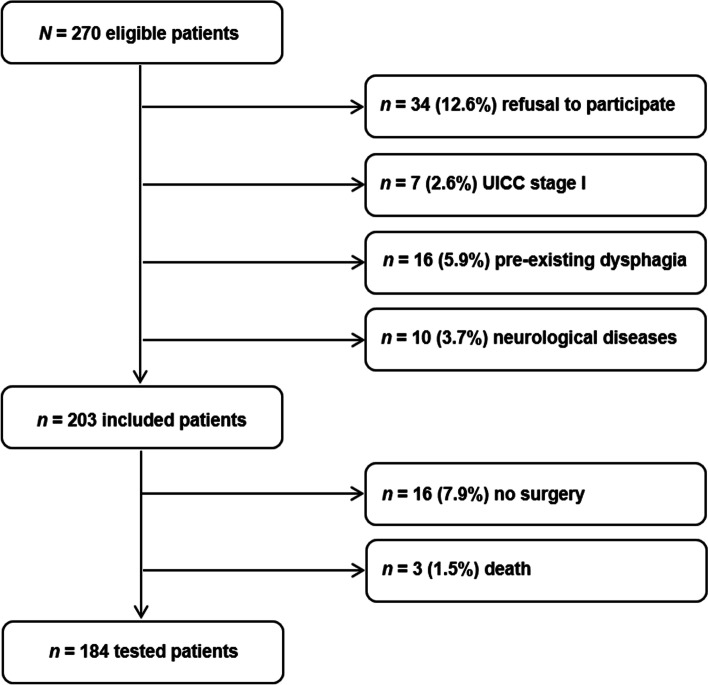
Eligibility flow chart. *UICC* tumor stage classification according to the Union for International Cancer Control

Out of 270 recruited head and neck cancer patients, 34 refused to participate in the study, 7 were excluded due to UICC stage I, 16 due to the pre-existing dysphagia, and 10 due to neurological diseases. Further 16 patients did not appear to surgery and 3 patients died prior to the WST (see Fig. 2).

A total of 184 patients were included in this study. On average, patients were 61.6 ± 10.3 years old (age range 18–88 years). The time span between surgery and patients’ participation in the study varied between 0 and 51 days (*M* = 14.8 ± 10.3). At this time point, all patients were still hospitalized, 48 (26.1%) of them had Percutaneous Endoscopic Gastrostomy (PEG) and 80 (43.5%) nasogastric tubes. For further patients’ characteristics, see Table 1.

**Table 1:** Patient characteristics and prevalence of relevant oropharyngeal dysphagia (OD), aspiration (ASP), and limitations of oral intake (LOI)

Patient characteristics		OD	ASP	LOI
	*n *(%)	*n *(%)	*n *(%)	*n *(%)
Biological sex				
Male	130 (70.7)	87 (66.9)	63 (48.5)	72 (56.2)
Female	54 (29.3)	33 (61.1)	17 (31.5)	30 (55.6)
Age (years)				
< 70	149 (81.0)	94 (63.1)	60 (40.3)	82 (55.0)
≥ 70	35 (19.0)	26 (74.3)	20 (57.1)	21 (60.0)
UICC tumor stage				
II	46 (25.0)	22 (47.8)	11 (23.9)	17 (37.0)
III	30 (16.3)	16 (53.3)	7 (23.3)	14 (46.7)
IV	108 (58.7)	82 (75.9)	62 (57.4)	72 (66.7)
Tumor site				
*Cavitas oris*	67 (36.4)	42 (62.7)	23 (34.3)	39 (58.2)
Oropharynx	93 (50.5)	66 (71.0)	47 (50.5)	55 (59.1)
Hypopharynx/larynx	24 (13.0)	12 (50.0)	10 (41.7)	9 (37.5)
Total	184 (100.0)	103 (56.0)	80 (43.5)	64 (34.8)

*UICC* tumor classification according to the Union for International Cancer Control

### Test procedure

After surgeon’s approval of and before the first postsurgical oral intake, all patients underwent the WST as screening tool and Fiberoptic Endoscopic Evaluation of Swallowing (FEES^®^) according to Langmore [23, 24] as the reference standard.

The WST was administered to patients in upright position by one of seven speech and language pathologists, all trained in performing the WST and most of them with at least five years’ experience in swallowing disorders. The duration of the WST was about 10 min.

FEES^®^ was performed within one hour after completing the WST by two phoniatricians with at least 10 years’ experience in FEES^®^. A transnasal flexible endoscope 11,101 RP2 (Karl Storz GmbH, Germany) was used for the examination, the records were done with an ENT video endoscopy system EndoStrob-DX (Xion medical GmbH, Germany). The examination took around 20 min and included water (2, 5, 10, and 20 ml), purée (2 and 5 ml) as well as a bite of cookie for testing solid consistency. The procedure was discontinued if aspiration occurred and was unchangeable by therapeutic intervention.

The FEES^®^ results were quantified by two scales, the Penetration aspiration scale (PAS) by Rosenbek et al. [25] and the Functional oral intake scale (FOIS) by Crary et al. [26]. A score of ≥ 6 in PAS defined aspiration (6 = material enters the airway, passes below the vocal folds, and is ejected into the larynx or out of the airway), a score of ≤ 4 in FOIS relevant limitations in oral intake (4 = total oral diet of a single consistency). A relevant oropharyngeal dysphagia was defined by linking these two scales so that patients with PAS ≥ 4 (4 = material enters the airway, contacts the vocal folds, and is ejected from the airway) or FOIS ≤ 4 were classified as having a relevant OD. Because a FOIS score of 4 did not occur in the sample, the cut-off value was de facto < 4 (tube dependency). OD was defined as the most important reference standard for the WST, PAS and FOIS as secondary reference standards.

### Statistical analyses

#### Descriptive statistics and influence factors on dichotomized FEES^®^ results

The prevalence of aspiration (PAS), limitations of oral intake (FOIS), and relevant oropharyngeal dysphagia (OD) was evaluated by descriptive statistics. The influence of tumor site, stage, patients’ age (18–69 vs. 70 + years) and biological sex on dichotomized FEES^®^ results (PAS, FOIS, and OD) was quantified by three binary logistic regressions (method “enter”). The results of regressions were of importance for the analysis of the representativeness of the study sample in comparison with all-German data on head and neck cancer, see Discussion.

#### Quality criteria of WST

Sensitivity (SENS), specificity (SPEC), positive likelihood ratio (LR+), and efficiency (EFF: percentage of test results including true positive and true negative ones correctly identified by WST) were determined for the dichotomized WST result (pass/fail). For this purpose, WST result (pass/fail) was cross-tabulated with dichotomized FEES^®^ results (PAS, FOIS, OD) as the reference standard.

#### WST item analysis and finalization of *FraMaDySc*

The following analyses aimed to derive the final version of *FraMaDySc* from the original WST and to improve its quality criteria. Items or failure criteria with a limited prediction power for OD as the main reference standard were to be identified and excluded. For this item analysis, phi-correlations between summarized “pass/fail” decisions for each volume (2, 5, 10, and 20 ml) and dichotomized OD according to FEES^®^ were calculated. Additionally, optional swallow attempts 1c and 2c as well as three WST failure criteria—wet voice before and after swallowing, cough/throat clearing after swallowing—were phi-correlated with dichotomized OD results.

All statistical analyses were conducted in the software package IBM SPSS 21 (International Business Machines Corp., New York, USA).

## Results

Aspiration occurred in 80 out of 184 patients (43.5%), including 33 (17.9%) with a silent aspiration (PAS grade 8). According to FOIS, limitations of oral intake were found in 103 patients (56.0%). OD was detected in 120 patients (65.2%).

In all three binary logistic regressions, the factor “UICC stage” showed a significant influence on the dependent variables: aspiration (*β* = 0.845, Wald = 16.25, *p* < 0.001), limitations of oral intake (*β* = 0.675, Wald = 13.10, *p* < 0.001), and OD (*β* = 0.670, Wald = 12.72, *p* < 0.001). Higher UICC grades were more closely associated with “fail” results than lower UICC grades (see Table 1 for descriptive statistics). Additionally, age 70 + years (*β* = 0.915, Wald = 4.70, *p* = 0.030) and male sex (*β* = 0.796, Wald = 4.72, *p* = 0.030) were associated with higher PAS grades. Chosen factors predicted about two thirds of the dichotomized dependent variables PAS (67.9%), FOIS (64.7%), and OD (66.8%).

The WST was failed by 119 out of 184 patients (64.7%). For the screening quality criteria of the WST, see Table 4. With 91–94%, WST SENS can be described as very good for all three dichotomized FEES^®^ results (PAS, FOIS, OD). SPEC delivered a good result only in case of OD (84%). The same is valid for LR+ (5.8) and EFF (89%).

In order to improve WST quality criteria, an item analysis was performed. Results of all WST water volumes were significantly associated with OD (see Table 2). Also, significant associations with OD were found for both optional c-swallow attempts, in spite of low sample sizes (1c: *φ* = 0.467, *p* < 0.001, *n* = 67; 2c: *φ* = 0.477, *p* = 0.049, *n* = 17). However, only one out of three failure criteria—cough/throat clearing after swallowing—yielded significant phi-correlations with OD in all four calculations (see Table 3). Voice quality after swallowing was significantly associated with OD in three out of four cases. Voice quality before swallowing did not show any significant results, except for a marginally significant result (*p* = 0.08) in the first swallow attempt. Because only “pass” results occurred in three out of four items of this failure criterion (5, 10, and 20 ml), phi-correlations could not be calculated.

**Table 2:** Associations of swallow attempts 2, 5, 10, and 20 ml with oropharyngeal dysphagia: phi-correlations (*φ*)

Volumes (ml)	“Fail” *n* (%)	*φ*
2	63/184 (34.2)	0.431
5	30/121 (24.8)	0.531
10	16/91 (17.6)	0.446
20	10/75 (13.3)	0.493

All* p* < 0.001

**Table 3:** Item reduction of the water swallow test: phi-correlations (*φ*) between failure criteria and oropharyngeal dysphagia as a reference standard

Volumes		Voice quality before swallow	Voice quality after swallow	Cough / throat clearing after swallow
ml	*n*	*φ*	*φ*	*φ*
2	176	0.120	0.400***	0.421***
5	119	–	0.364***	0.494***
10	89	–	0.326**	0.506***
20	75	–	0.213	0.494***

***p < 0.001, **p < 0.01

Thus, according to phi-correlations between the WST and OD, several WST items appeared redundant: all four items on the voice quality before swallowing and voice quality after swallowing of 20 ml. However, the item on the voice quality before the first swallow attempt (2 ml) showed a marginally significant association with OD and constituted an independent WST failure criterion (see “Methods”). Therefore, this item was retained. Also, the item on the voice quality after swallowing of 20 ml was not excluded in order to make the WST structure more uniform and homogenous (in analogy with the voice quality after swallowing of other three volumes). Thus, only three items were deleted from the WST for the finalization of *FraMaDySc*: voice quality before swallow attempts of 5, 10, and 20 ml (for the final version of *FraMaDySc*, see Supplement 1). In comparison with the first WST version, specificity, positive likelihood ratio, and efficiency increased for OD as the main reference standard (see Table 4), with sensitivity remaining on the same level.

**Table 4:** Quality criteria of the original WST and finalized *FraMaDySc* for oropharyngeal dysphagia (OD), aspiration (ASP), and limitations of oral intake (LOI)

Reference standards	Results (tp/fp/fn/tn)	Sensitivity (%) 95% CI	Specificity (%) 95% CI	LR+ 95% CI	Efficiency 95% CI
Original water swallow test					
OD	109/10/11/54	90.8 (84.2 – 95.3)	84.4 (73.1 – 92.2)	5.8 (3.3 – 10.3)	88.6 (83.1 – 92.8)
ASP	75/44/5/60	93.8 (86.0 – 97.9)	57.7 (47.6 – 67.3)	2.2 (1.8 – 2.8)	73.4 (66.4 – 79.6)
LOI	94/25/9/56	91.3 (84.1 – 95.9)	69.1 (57.9 – 78.9)	3.0 (2.1 – 4.1)	81.5 (75.2 – 86.9)
*FraMaDySc*					
OD	109/8/11/56	90.8 (84.2 – 95.3)	87.5 (76.9 – 94.5)	7.3 (3.8 – 13.9)	89.7 (84.3 – 93.7)
ASP	75/5/42/62	64.1 (54.7 – 72.3)	92.5 (83.4 – 97.5)	8.6 (3.7 – 20.2)	74.5 (67.5 – 80.6)
LOI	94/9/23/58	80.3 (72.0 – 87.1)	86.6 (76.0 – 93.7)	6.0 (3.2 – 11.1)	82.6 (76.3 – 87.8)

*tp* true positive, *fp* false positive, *fn* false negative, *tn* true negative, *LR+* positive likelihood ratio, *CI* confidence interval

## Discussion

The results presented here underscore oropharyngeal dysphagia as a relevant postsurgical consequence in head and neck cancer patients. Two-thirds of all patients are affected, with a high prevalence of aspiration (44%) and limitations of oral intake (56%). This emphasizes the necessity to identify these postsurgical sequels and to scrutinize potential influence factors in this specific patient population. Among the analyzed factors, only higher UICC stages were associated with a risk of oropharyngeal dysphagia, which supports findings of previous studies [6]. The same applies to limitations of oral intake [27]. Aspiration was influenced by three factors. First, it was significantly affected by a higher patients' age, which supports the findings of Jung et al. [28] and Xu et al. [8]. The other two influence factors were male sex, also described by Xu et al. [8], and, again, higher tumor stages [27, 29].

The purpose of the study was to develop *FraMaDySc* as a valid screening tool for the convenient, quick, and easy identification of head and neck cancer patients with a relevant postsurgical oropharyngeal dysphagia as well as with aspiration and limitations in oral intake. This goal has been achieved: The quality criteria are remarkable at least for the detection of a relevant oropharyngeal dysphagia as the main reference standard, with good sensitivity (91%), specificity (88%), and positive likelihood ratio (7.3). However, the quality criteria for predicting the secondary reference criteria, the aspiration (SENS 64%, SPEC 93%, LR+ 8.6) and limitations of oral intake (SENS 80%, SPEC 87%, LR+ 6.0), are less reliable. In other words, swallowing of water, that is, of only one consistency in *FraMaDySc* showed a limited predictive power for the aspiration and limitations of oral intake of three consistencies that were tested in FEES^®^ (water, purée, and cookie).

Although some other WST were developed and validated for head and neck cancer patients, their target population did not include postsurgical patients and their reference standard was limited to aspiration [21, 22]. Therefore, *FraMaDySc* is not directly comparable to other screenings aiming at the identification of swallowing problems. Among all WST, the Volume-Viscosity Swallow Test (V-VST) might be most comparable with *FraMaDySc* although the validation sample contained many other etiologies apart from head and neck cancer [30]. With the sensitivity of 93% and specificity of 81%, the quality criteria were comparable to those of *FraMaDySc*. However, available data do not allow to draw any conclusion on V-VST quality criteria in postoperative head and neck cancer patients. For the identification of aspiration in postoperative head and neck cancer patients, the combination of WST with a food test by Horii et al. [31] might be a better choice than *FraMaDySc* with a sensitivity of 100% and specificity of 73%. However, a very limited sample size of *N* = 36 and an alternative PAS dichotomization (“fail” = PAS ≥ 3 instead of ≥ 4 in the study presented here) should be taken into account. Another screening tool for head and neck cancer patients is the 100 ml WST by Patterson et al. [22]. However, it is not directly comparable with *FraMaDySc* because it was designated to identify aspiration instead of OD and the target population comprised head and neck patients who underwent radio(chemo)therapy instead of surgery. The quality criteria of both *FraMaDySc* (SENS 64%, SPEC 93%) and 100 ml WST by Patterson, for instance three months after the therapy onset (SENS 77%, SPEC 47%), for predicting aspiration were not optimal. Also, positive likelihood ratio of the 100 ml WST by Patterson was very low (1.7) and close to the value 1 that is achieved by coin tossing. On the contrary, LR+ 8.6 of *FraMaDySc* means a moderate increase in the likelihood of disease (a large increase begins with LR+ 10) and, thus, is much more reliable in the detection of patients with aspiration. Because *FraMaDySc* represents the very first screening developed for postsurgical head and neck cancer patients, this LR+ can be considered satisfactory. The Brief Bedside Dysphagia Screening Test-Revised (BBDST-R) [32] was developed for a mixed population including some head and neck cancer patients and achieved a sensitivity of 96% and specificity of only 26% for the prediction of the PAS result, however, with yet another PAS dichotomization (“fail” = PAS ≥ 2).

In course of the WST item reduction in the present study, all four water volumes contributed significantly to the prediction of swallowing difficulties after surgery. Therefore, none of the volumes could be excluded from the WST. Among WST failure criteria, *voice quality before swallowing* was of a limited value for the prediction of OD. Only the item *voice quality before the first swallow* turned out to be marginally significantly associated with the oropharyngeal dysphagia. This significant result can be explained by the failure criterion according to which the test was terminated before the first swallow attempt if the patient could not clear his/her voice. In such cases, this item was the only one that was filled out in the WST form. Among items on the voice quality after swallowing, those after 2, 5, and 10 ml were of importance for the prediction of OD. Item on the voice quality after the swallow attempt of 20 ml did not yield a statistically significant result. However, low sample sizes in the last WST item should be considered in this case. The item on the voice quality after the swallow attempt of 20 ml was not deleted in order to standardize the *FraMaDySc* test procedure and to reduce performance inaccuracy.

The representativeness of the study sample can be confirmed for the age, biological sex, and tumor stage of patients. Thus, according to the data of the German Robert Koch Institute [33], head and neck cancer patients in Germany were, on average, above 60 years old, much more often male then female, and had UICC stage IV (under the condition of exclusion of stage I, as was done in the present study). No comparison regarding tumor localization is possible because Robert Koch Institute subdivides head and neck cancer into two categories only: larynx (18% of all head and neck cancer cases in terms of incidence) and oral cavity/pharynx (82%). However, a very large proportion of oropharynx cancer in the present sample (51%) might be a limitation of the study. Also, larynx and hypopharynx cancers had to be managed as one group due to their low frequency. However, because the factor “tumor site” had no influence on the dichotomized FEES^®^ results in all three regressions, these limitations were of no relevance for the development of *FraMaDySc*.

Further research is needed to quantify *FraMaDySc* reliability as well as quality criteria before and longer time after surgery. For stroke patients, dysphagia screenings showed a significant protective health benefit for pneumonia, mortality, tube dependency, and length of stay in a hospital [34]. In future research, benefits of *FraMaDySc* should be analyzed in this respect. Also, higher sample sizes are necessary to differentiate between larynx and hypopharynx cancer or between patients with different surgical techniques and extents of reconstruction. Because dysphagia to liquids is frequent after partial laryngectomy but does not have the same significance as after oropharyngectomy, a comparison of these two tumor sites (larynx vs. oropharynx) should be carried out regarding the predictive power of *FraMaDySc* for FEES^®^ results.

*FraMaDySc* provides not only good quality criteria for the identification of oropharyngeal dysphagia but also fulfills other important requirements for a screening tool. *FraMaDySc* can be considered a simple and feasible test. Due to the successful item reduction, the test was even shortened from 20 to 17 items with reducing the administration time from 10 to 8 min. The test performance and the failure criteria are standardized, which minimizes their subjectivity. Increasing volumes of water reduce the risk of aspiration and the failure criteria (wet voice and cough) assure the patient safety. Taking into account good to excellent quality criteria (sensitivity, specificity, likelihood ratio), feasibility, standardization, and safety of this WST, *FraMaDySc* can be considered an excellent screening tool for the identification of a relevant oropharyngeal dysphagia in head and neck cancer patients undergoing surgery. It is recommended that this screening is conducted in postsurgical head and neck cancer patients after surgeon’s approval of the first postsurgical oral intake. In case of the “fail” result, FEES^®^ should be performed due to a high risk of oropharyngeal dysphagia. In case of the “pass” result, the oral intake can be resumed without FEES^®^.

## Supplementary Information

Below is the link to the electronic supplementary material.

Supplementary file1 (DOCX 29 KB)

## Data Availability

The data are not publicly available due to ethical restrictions.
