# Supplementary material for: FraMaDySc: dysphagia screening for patients after surgery for head and neck cancer
Source: Eur Arch Otorhinolaryngol. 2023 Feb 14;280(5):2585–92. doi: 10.1007/s00405-023-07865-6 (PMC10066133; doi:10.1007/s00405-023-07865-6)
Supplement: Supplementary file 1 — Supplementary file1 (DOCX 29 KB) [file 405_2023_7865_MOESM1_ESM.docx]

**Supplement 1: FraMaDySc**

***FraMaDySc***

**“Frankfurt/Marburg Dysphagia Screening”**

**for patients with head and neck cancer after surgery**

Patient’s name: ________________________________________ Birth date: ____________

Date: __________ Tester: ______________________________________ ID: _________

**□** Wet voice before the first swallow (🡪 fail)

**Swallow attempts: 2 ml still water by a teaspoon**

|  | **1a swallow** | **1b swallow** | **1c swallow** |
| --- | --- | --- | --- |
| **Voice quality after swallow** | □ pass □ fail | □ pass □ fail | □ pass □ fail |
| **Cough or throat clearing** | □ pass □ fail | □ pass □ fail | □ pass □ fail |

**Swallow attempts: 5 ml still water by a beaker**

|  | **2a swallow** | **2b swallow** | **2c swallow** |
| --- | --- | --- | --- |
| **Voice quality after swallow** | □ pass □ fail | □ pass □ fail | □ pass □ fail |
| **Cough or throat clearing** | □ pass □ fail | □ pass □ fail | □ pass □ fail |

**Swallow attempts: 10 and 20 ml still water by a beaker**

|  | **3a swallow (10 ml)** | **3b swallow (20 ml)** |
| --- | --- | --- |
| **Voice quality after swallow** | □ pass □ fail | □ pass □ fail |
| **Cough or throat clearing** | □ pass □ fail | □ pass □ fail |

***FraMaDySc* result:** □ pass □ fail

***FraMaDySc* failure criteria:**

(a) wet voice before the swallow attempt 1a

(b) wet voice or voice change after swallowing (all swallow attempts)

(c) cough or throat clearing after swallowing (all swallow attempts)
